# Early Detection and Management of *Lamanema chavezi* infection in a llama *(Lama glama*) in Switzerland

**DOI:** 10.1007/s11259-024-10465-2

**Published:** 2024-07-16

**Authors:** Diana S. Gliga, Anne Kramer, Gastón Moré, Caroline F. Frey, Walter Basso

**Affiliations:** 1 Institute of Parasitology, Vetsuisse Faculty, University of Bern, Bern, Switzerland; 2 Alpakavet, Uettligen, Switzerland

**Keywords:** *Lama glama*, Switzerland, *Lamanema chavezi*, Strongylid, Hepatitis, Eprinomectin

## Abstract

*Lamanema chavezi *is an entero-hepatic strongylid parasite specific to South American camelids. It has been reported only on few occasions outside South America. Due to its hepatic migration, it can cause extensive liver damage, leading to granulomatous and fibrotic hepatitis and manifesting with lethargy, anorexia, and even death. We are reporting the second case of *L. chavezi* infection in Europe and the first in Switzerland. The patient was a three-year old neutered male llama (*Lama glama*). Clinical examination revealed bloody mucous discharge from the anus. Fecal sedimentation/flotation revealed strongylid eggs consistent with *L. chavezi*, which were molecularly confirmed by a PCR targeting the ITS2 plus 5.8S and 28S rDNA flanking regions and amplicon sequencing. Eighteen weeks after administration of a single dose of eprinomectin (0.2 mg/kg i.m.), no further *L. chavezi* eggs were detected in the feces. The source of infection could not be traced back. The entire herd consisted of llamas bred in Switzerland. *L. chavezi* has been rarely reported outside South America, but its potential for pathogenicity and establishment should not be underestimated. Fecal sedimentation/flotation techniques should be routinely performed to ensure early detection of the parasite.

## Introduction

*Lamanema chavezi* (Becklund 1963) is a monoxenous strongylid nematode of the family Molineidae, native to the South American continent (Rickard and Hoberg 2000). It was first described in the small intestine of alpacas (*Lama pacos)* and vicuñas (*Lama vicugna)* in Peru as a monotypic genus (Becklund 1963) and later redescribed by Angulo et al. (2015). This is one of the most pathogenic nematodes which may affect domestic (llama [*Lama glama*] and alpaca) and wild (vicuña and guanaco [*Lama guanicoe*]) South American camelids (SAC) (Bauer et al. 2024). Adult parasites inhabit the small intestine and release eggs into the environment with the feces. Like *Nematodirus* spp., *L. chavezi* larvae require a long time to develop up to the third-stage stage (L3), which stays inside the eggshell until a freeze–thaw stimulus in the pastures triggers their hatching. After ingestion, L3 travel from the small intestine via the blood to the liver, where they mature into fourth-stage larvae (L4), and migrate through the parenchyma to the bile ducts, from where they return to the small intestine to develop further into adult worms and release eggs 30–32 days later (Rojas et al. 1986; Bauer et al. 2024). The hepatic migration is of particular interest, since it is responsible for important liver damage consisting of hemorrhages, necroses, mineralizations, and abscesses. Clinical signs of infection include anemia, anorexia, emaciation, recumbency, and even death (Cafrune et al. 2001; Jarvinen et al. 2014; Bauer et al. 2024). *L. chavezi* infection negatively affects SAC production by liver condemnation and reduced carcass quality. In one slaughterhouse in Peru, 37.7% of livers from all alpacas (n = 7,888) slaughtered from January to August 2018 were condemned due to *L. chavezi* infection (Huamaní 2019). The prevalence of *L. chavezi* eggs in two alpaca populations from Peru was 0.7% (n = 1,319) (Contreras et al. 2014). *L. chavezi* infections were also reported in SAC in Chile, Bolivia, Ecuador, and Argentina, additionally to Peru (Cañal and Beltrame 2022).

Domestic SAC are exported worldwide and bred locally as exotic species. Therefore, some of their host-specific parasites were towed along and became endemic in the new areas e.g., SAC-specific *Eimeria* spp.. Imported parasites may be a threat to locally bred SAC. So far, *L. chavezi* has been detected outside its natural range in USA (Jarvinen et al. 2014), New Zealand (McKenna et al. 2009), and more recently in Germany, causing the death of a 15-month-old male and a 3-year-old female llama in the years 2017 and 2018, respectively (Bauer et al. 2024). Here we present a further case of *L. chavezi* infection in Europe, namely in a llama in Switzerland, with a mild disease course.

## Material and methods

A three-year-old neutered male llama with classic coat type was part of a herd of nine females and 16 further neutered males, aged from six months to 19 years, most animals ranging from nine to 15 years old. The herd had open access to a pasture, shared with horses and donkeys. Llamas were kept for animal-assisted therapy and commercial trekking. All llamas were purchased from Swiss breeders, and all were born in Switzerland. The patient was two years old when purchased. The farm was located at the foothills of the Jura mountains in the Canton of Bern, Switzerland (510 m.a.s.l., annual rainfall 2000–2022: 1256 ± 143 mm, Cfb climate zone). On 25th July 2023 the patient presented bloody mucous discharge from the anus. A fecal sample was immediately sent to the diagnostic laboratory of the Institute of Parasitology at the Faculty of Veterinary Medicine in Bern. Standard coprological techniques for examination of SAC feces were conducted: combined sedimentation/flotation technique (zinc chloride 44%, specific gravitiy 1.3 and zinc chloride 66%, specific gravitiy 1.45), sedimentation and McMaster egg counting with a detection limit of 50 epg (saturated NaCl solution, specific gravity 1.2). In addition, three pooled fecal samples from the llama herd were subsequently examined. For molecular confirmation, the flotation sample (sugar solution of specific gravity 1.3) under the coverslip was washed into a small Petri dish and 25 *Lamanema*-like eggs were transferred under stereomicroscope with a pipette into an Eppendorf tube prefilled with 0.5 ml water. DNA was extracted from these eggs using a commercial kit (Quick-DNA Fecal/Soil Microbe, Zymo Research, USA). Subsequently, a PCR targeting the ITS-2 plus 5.8S and 28S rDNA flanking regions was performed using the nematode universal primers NC1 (forward, 5 ‘-ACGTCTGGTTCAGGGTTGTT -3 ‘) and NC2 (reverse, 5 ‘-TTAGTTTCTTTTCCTCCGCT-3 ‘) (Gasser et al. 1993). The amplicons were visualized by gel electrophoresis, purified (DNA Clean & Concentrator™-5, Zymo Research, USA), and submitted for Sanger sequencing to an external laboratory (Microsynth AG, Balgach, Switzerland). Larval culture with a modified regimen (incubation over four weeks at room temperature (RT)– two days cold shock at 4°– two weeks at RT) was performed. Liver ultrasonography was performed to check for related changes.

## Results

**Fig. 1:**
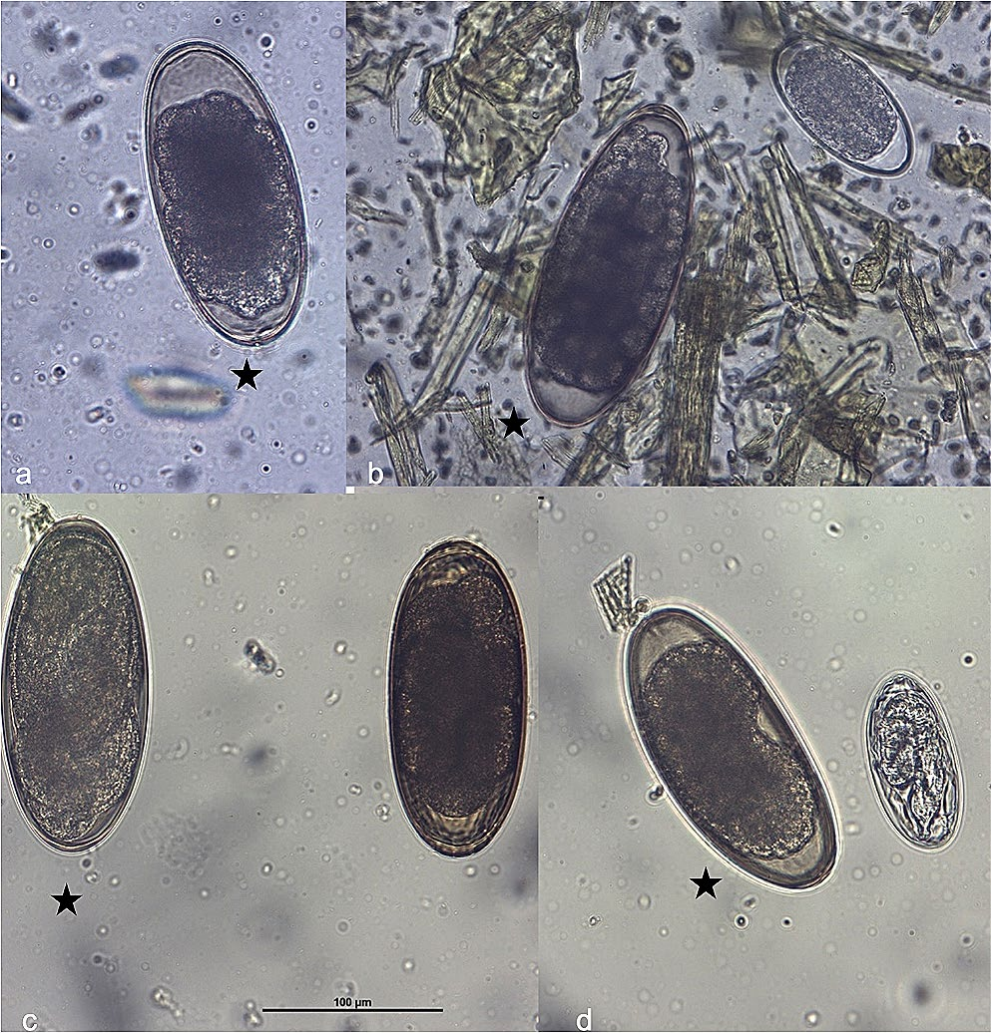
*Lamanema chavezi* eggs (marked with star) in different developmental stages and compared with other strongylid eggs. (**a**) *L. chavezi* with slight thickenings at the poles and poorly distinguishable small blastomeres; (**b**) *L. chavezi* egg with numerous distinguishable small blastomeres, compared to a common strongylid egg; (**c**) embryonated* L. chavezi* egg compared with a *Nematodirus battus* egg; (**d**)* L. chavezi *egg with poorly distinguishable blastomeres compared with an embryonated common strongylid egg

**Fig. 2:**
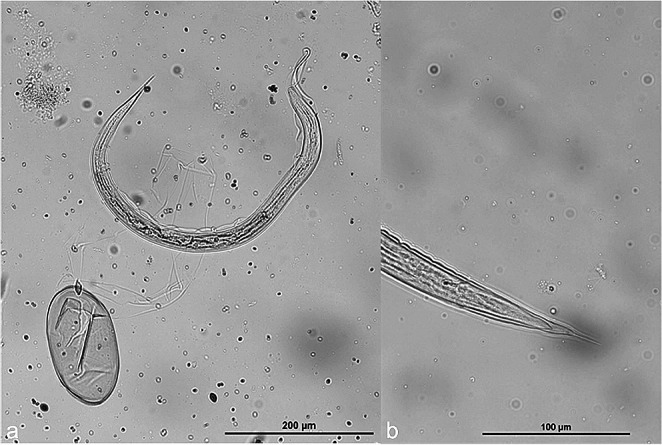
(**a**) *Lamanema chavezi* L3 freshly hatched from the egg after applying pressure on the coverslip, remaining eggshell is visible; (**b**) Posterior end of *Lamanema chavezi* L3

Overall, eggs of following parasites could be identified: *Nematodirus battus*, *Strongyloides* sp., *Dicrocoelium dendriticum* and strongyle-type eggs (800 epg). In addition, eggs resembling those of *L. chavezi* were noticed. These eggs were large, thick-shelled, elliptic, with multiple small-sized blastomeres and air chambers at both poles. Length and width of 13 eggs were measured. Mean length ± standard deviation was 177.7 ± 8.8 mm (range 168.3–197.2 mm) and width 79.1 ± 3.3 mm (range 72.8–97.6 mm) (Fig. 1). The three pooled fecal samples from the herd were positive for *Trichuris* sp. eggs, gastrointestinal strongylids, and *N. battus*. No further *L. chavezi* eggs were identified in the samples at that timepoint, but a follow-up examination of the whole herd is planned. Since *L. chavezi* had not been previously reported in Switzerland, we performed additional tests for confirmation: coproculture, PCR, and sequencing. The obtained sequence (primers trimmed) was 100% identical (249/249 bp) to GenBank sequences of *L. chavezi* (Accession no. MG598428, MG598425, MG598420, MG598418) and was deposited under accession no. PP060927. Larval culture yielded embryonated *L. chavezi* eggs and few free larvae of poor viability. When the larvated eggs were pressed with a needle under the microscope, a sheathed larvae emerged having eight intestinal cells and a pointed posterior end (Fig. 2).

**Fig. 3:**
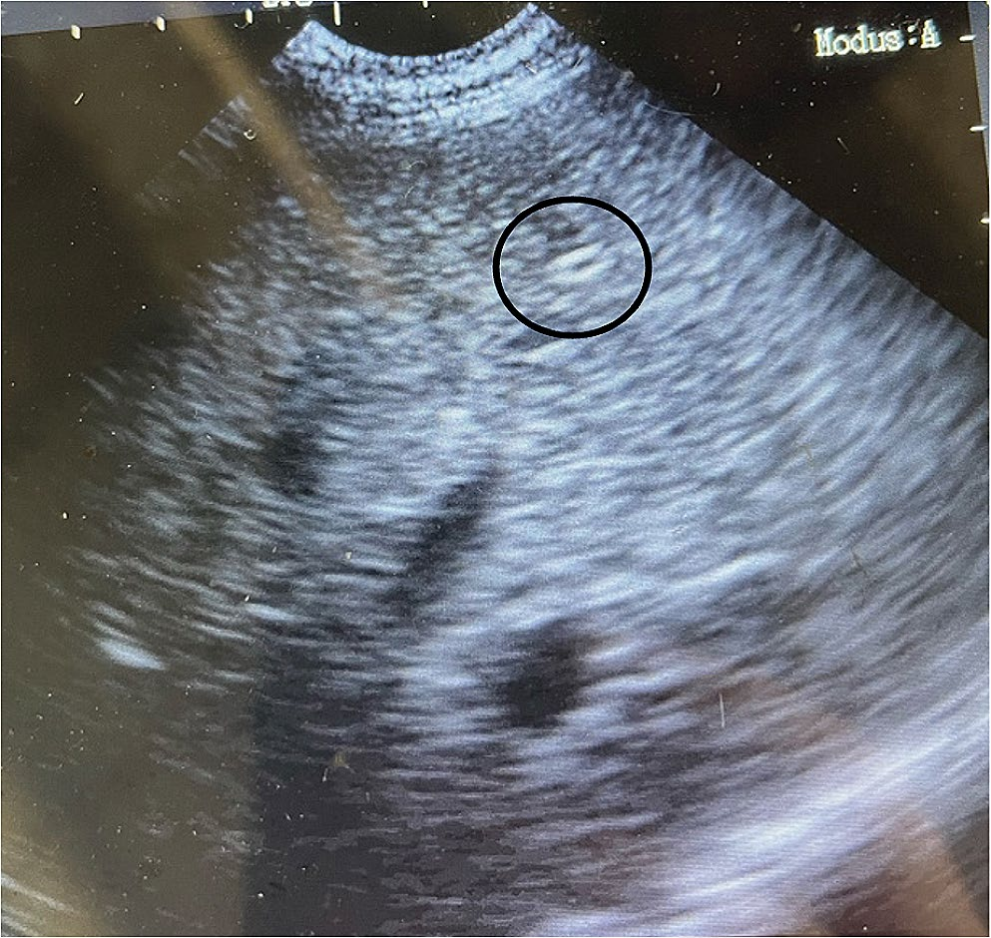
Transabdominal ultrasound image of the liver with hyperechoic structure (encircled) without acoustic shadows; represents thickening of the bile duct without calcification

The infected llama was treated once with eprinomectin 0.2 mg/kg i.m. A fecal examination 16 days post-treatment showed few deformed common strongylid and *L. chavezi* eggs (< 50 epg). In December 2023 (4.5 months after therapy) the fecal exam was positive only for *D. dendriticum* and *Trichuris* sp. eggs. At the time of diagnosis, the liver had by ultrasound a normal aspect apart from one hyperechoic point structure that could not be investigated further (Fig. 3).

## Discussion

We report the presence of *L. chavezi* in Switzerland. To our knowledge, this is the second report of *L. chavezi* in Europe. Previous surveys of SAC endoparasites in Switzerland and neighboring countries could not detect *L. chavezi* (Switzerland: Hengrave Burri et al. 2005; Austria: Lambacher et al. 2016; Germany: Kultscher et al. 2019). The diagnosis of the first cases in Germany was made post-mortem in two llamas. Both animals died after a brief episode of diarrhea (case 1), or emaciation, apathy, anorexia, anemia and tetraparesis (case 2), and *L. chavezi* was suspected to be the cause of fatal disease in both animals (Bauer et al. 2024). The infected llama in Switzerland was in good body condition. The observed bloody mucous anal discharge could have been due to *L. chavezi*; however, this is a non-specific clinical sign, which may also be caused by other parasite species present, such as *Trichuris* sp, or by unrelated pathogens such as bacteria (Konieczny and Pomorska-Mól 2023). Moreover, since the animal also harbored *D. dendriticum*, the liver functionality could be impaired by a potential synergic effect of both parasites. For morphological identification of *L. chavezi* eggs, fecal samples should be fresh, otherwise, they can be confused with those of *Nematodirus* spp. after embryonation begins. Cultivation of third stage larvae of *Nematodirus* spp. and *L. chavezi* is more difficult compared to common strongylids because of the prolonged duration and the need for a cold or freezing stimulus. Culture conditions vary across different studies and the timing of the cold stimulus seems to be critical for hatching (Jarvinen et al. 2014; Angulo et al. 2015). Although possible, it is not clear whether the focal lesion observed by ultrasonography was related to *L. chavezi* infection. Highly echoic bile ducts despite homogeneous liver parenchyma may be indicative of calcification caused by *D. dendriticum* (Wenker et al. 1998) and this parasite was also present. Additional parasitic hepatic disease in SAC such as fascioliasis (Hamir and Smith 2002), cystic echinococcosis (Sanchez et al. 2022) and cysticercosis due to *Taenia hydatigena* (Leguía and Casas 1999) must also be taken in consideration. However, in the present case, *Fasciola hepatica* eggs were not detected by the sedimentation method and Switzerland is considered non-endemic for *Echinococcus granulosus* given the lack reports of locally acquired cystic echinococcosis in humans or animals (Deplazes et al. 2017; Casulli et al. 2022) and absence of *E. granulosus* eggs in canids from Switzerland (Frey et al. 2010; Nagy et al. 2011; Hauser et al. 2015).One sole treatment with eprinomectin appeared to clear the patent infection in this llama. Long-term surveillance of fecal parasites is needed to confirm therapy success or identify a possible reinfection. Another successful treatment may be levamisole (8 mg/kg BW orally) as this resulted in 96% fecal egg count reduction (FECR) in llamas (Gillespie et al. 2010). Although *L. chavezi* is considered a SAC-specific parasite, adult male and female *L. chavezi* worms were reported in the South American rodents *Lagidium viscacia* (Sutton and Durette-Desset 1985) and *Phyllotis* sp. (Digiani and Durette-Desset 2007) based on morphological features. These studies did not provide information on *L. chavezi* pathogenicity in these likely aberrant hosts. Molecular characterization of stored specimens could provide clarification whether the genus *Lamanema* is indeed monotypic or not. Spill-over of *L. chavezi* into European rodents could potentially contribute to the spread of *L. chavezi;* however, no data on this possibility are available so far. SAC can suffer from possibly lethal parasitic infections by different modes. On one hand, naïve SAC outside their original habitat may be exposed to parasites endemic in other regions (e.g., Europe) such as *Dicrocoelium dendriticum* with important clinical consequences (Wenker et al. 1998). On the other hand, imported parasites can spill-over to SAC bred outside South America, potentially establishing in new geographic regions, such the case of *Nematodirus lamae* infection in an alpaca bred in the UK (Mitchell et al. 2016).

Current records from Switzerland show a total of 6676 registered SAC in 2022 (i.e., 3808 alpacas and 2868 llamas, with 491 and 314 farms keeping alpacas and llamas, respectively, and increasing numbers over the last 20 years (Federal Statistical Office of Switzerland 2023). Import from South America and trade among different European and non-European countries is being practiced; therefore, further introduction of *L. chavezi* or other SAC-specific parasites cannot be excluded and a quarantine period with coproscopical testing are advisable. Regular screening for gastrointestinal parasites can aid in the early detection of *L. chavezi* and reduce the infection pressure on the pasture by treating patent animals. Diagnostic laboratories should be aware of the novel occurrence of this parasite and familiarize themselves with the morphology of *L. chavezi* eggs. *Lamanema chavezi* eggs are highly resistant in extremely cold climates (Rojas et al. 1986) therefore the life cycle could easily establish on alpine pastures in Switzerland,

## Data Availability

DNA sequence is deposited in GenBank (no. PP060927).
